# Supplementary material for: MOSAIC (MOthers’ AdvocateS In the Community) for pregnant women and mothers of children under 5 with experience of intimate partner violence: A pilot randomized trial study protocol
Source: PLoS One. 2022 May 18;17(5):e0267679. doi: 10.1371/journal.pone.0267679 (PMC9116623; doi:10.1371/journal.pone.0267679)
Supplement: S1 File — (DOCX) [file pone.0267679.s002.docx]

**Michigan State University Human Research Protection Program**

| - Complete this template for new exempt, expedited, or full board studies.   - Complete Section I for ALL studies (exempt, expedited, full board)   - Complete Section II ONLY if your study does not qualify for exemption and requires an expedited or full board review. Contact the IRB office if you have any questions. - CLICK™ IRB:   - Include the template with a New Study Submission.   - Upload the completed template to the Basic Information SmartForm page, Question 10.   - When uploading documents to Click (e.g. consent documents, instrument), provide distinct file names. - See the Click Quick Guides and the HRPP Manual for more information, available at hrpp.msu.edu | | | | | | | |
| --- | --- | --- | --- | --- | --- | --- | --- |
|  | | | | | | | |
| **Study Title:** | | | | | MOSAIC (MOthers' AdvocateS In the Community) for Pregnant Women and Mothers of Children Under 5 with Experience of Intimate Partner Violence | | |
| **Click Study ID (if known):** | | | | |  | | |
| **Sponsor (if applicable):** | | | | | NIMH | | |
| **Sponsor ID (if applicable):** | | | | |  | | |
|  | | | | | | | |
| **Section I. IRB Protocol for All Studies**  Section I is completed for ***all studies*** and includes questions to determine whether the study qualifies for exemption. Section II is only completed if the study does not qualify for exemption. | | | | | | | |
|  | | | | | | | |
| **1.** | **Hypothesis / Objective / Goals / Aims.** | | | | | | |
|  | Briefly describe the study’s hypothesis / objectives / goals / aims. | | | | | | |
|  | The purpose of this R34 exploratory research proposal is to conduct formative work for a larger randomized controlled trial (RCT) evaluating the effectiveness of the “Mother AdvocateS In the Community (MOSAIC) Plus” intervention to reduce depressive and PTSD symptoms and prevent additional IPV among pregnant women and mothers with children under 5 experiencing IPV. The MOSAIC Plus intervention will integrate IPT principles and skills into the MOSAIC intervention in order to expand it to address consequences of IPV, including depression and PTSD symptoms. The proposed study will enroll pregnant women who report experience of IPV in the past 6 months, and who screen positive for elevated depressive and/or PTSD symptoms. The intervention lasts 9 months after enrollment. Women will be recruited from Obstetrics and Gynecological unit of the Hurley Medical Center and YWCA, Flint. The control condition will be Enhanced Treatment as Usual (ETAU). Study assessments will take place at baseline, 3, 6 and 9 months.  The development aims of this proposal are to:  1. Enhance and expand the MOSAIC manual (designed to be delivered by lay providers) with lay-provider friendly IPT principles for reducing depressive and PTSD symptoms.  2. Refine the manual, intervention, and study procedures using 6 focus groups: 3 groups with mothers who have experienced IPV in the past 6 months (n~20), 2 with potential mentor mothers (n~16), and 1 group with nurse-family partnership staff (n~8)  3. Develop, implement, and evaluate the intervention training program.  4. Improve the clarity, content, acceptability, and feasibility of the enhanced MOSAIC Plus intervention through a small open trial (n = 15) of mothers with past 6-month IPV experience  The pilot study aims of this proposal are to:  5. Conduct a pilot RCT in a sample of 40 mothers with IPV experience in the past 6 months to demonstrate the feasibility and acceptability of the proposed recruitment methods, research design, intervention training methods, and of delivering the MOSAIC Plus and ETAU interventions. As recommended by NIMH, we will also examine 95% confidence intervals around differences between the proposed intervention and ETAU for the following outcomes through 9 months after baseline:  a. Mental health outcomes. Reduction in depressive (primary) and PTSD symptoms  b. IPV outcomes. Decreased subsequent IPV over the 9 months measured by CAS  c. Functioning outcomes. Improvement in functioning, self-care and general health and wellbeing  d. Potential target mechanisms. Increased social support and effectiveness obtaining resources. | | | | | | |
| **2.** | **Subject Population.** | | | | | | |
| **2A.** | Study purposefully includes the following subject population(s) (select all that apply): | | | | | | |
|  | Cognitively impaired adults  Minors (children) (view information about the definition of a child)  Minors who are wards of the state  Pregnant women, fetuses, or neonates  Prisoners  Students | | | | | | |
| **2B.** | Study involves (select all that apply): | | | | | | |
|  | Funding, support, or other requirement to comply with U.S. Department of Justice regulations  Incomplete disclosure or attempted deception of subjects | | | | | | |
|  | *CLICK IRB: Upload the debriefing script, document, etc. to the Consent Forms and Recruitment Materials SmartForm page, Question 1.* | | | | | | |
| **3.** | **Estimated Study Duration.** | | | | | | |
|  | Provide the time estimated to complete all human subject research, including analysis of the subjects’ identifiable private information. | | | | | | |
|  | 36 months | | | | | | |
| **4.** | **Reasonably Foreseeable Risks.** | | | | | | |
| **4A.** | There are (select one of the following): | | | | | | |
|  | No reasonably foreseeable risks to subjects  Reasonably foreseeable risks to subjects | | | | | | |
| **4B.** | Explain the selection. *If you selected that there are reasonably foreseeable risks to subjects, describe the risks, considering physical, psychological, social, legal and economic risks.* | | | | | | |
|  | There may be a potential risk of study related coersion. Interviews about mental health could be distressful to subjects. | | | | | | |
| **4C.** | If you selected that there are reasonably foreseeable risks, describe the procedures for protecting against or minimizing potential risks and provide an assessment of their likely effectiveness. | | | | | | |
|  | 1. We will minimize the risk of potential coercion by following standard procedures for obtaining informed consent. We will begin this process during the intake, where we will clarify the nature of the study and possible alternatives from the outset. Prior to enrolling women in the research, we will fully explain the study procedures, risks, benefits, and alternatives to women, emphasizing that the woman’s participation has no impact on the other services she receives at the community or at the health facility. Also, women who do not consent or who withdraw at any point during the study will receive appropriate referrals (e.g., mental health, perinatal care and/or substance use treatment), if needed. All women will be reminded that there is no penalty for women who choose to not participate or to withdraw from the study and that their decision to participate does not impact the standard services they receive through the OB unit of Hurley Medical Center, YWCA or at the community. All reimbursements for participating will be commensurate with participants’ time required for participating in the research.  2. We will minimize the risk of distress. Participants of this study (i.e. pregnant women and mothers with children under 5 with the experience of IPV, who also report elevated levels of depressive and PTSD symptoms) may face the risk of increased distress during assessment procedures, during the control condition, or during the experimental condition. All women will be informed that they do not have to answer questions that they find too distressing and will be reminded that they can discontinue participation at any time. Participants will be formally assessed at intake, 3 and 6 months follow ups. Participants who become upset during study interviews will be referred to Dr. Hailemariam or Dr. Johnson. All research personnel (i.e., mentor mothers and a research assistant) will be trained in the protocol for homicidal or suicidal risk and research procedures for these situations. Procedures for suicide ideation, homicide ideation, clinical deterioration, etc. can be found in the Data Safety and Monitoring Plan. All mentor mothers will be given a study cell phone.  Referrals for additional care. During the study, any participant who presents serious/acute physical or mental health problems will be promptly referred to the needed services. We have a list of free or low-cost medical care and mental health treatment clinics in Genesee County. If acute IPV services are needed, we will refer women to local domestic violence shelters. | | | | | | |
| **5.** | **Conflict of Interest.** | | | | | | |
|  | Do any investigators or research staff have a financial interest related to the research that has not otherwise been disclosed elsewhere in this submission? | | | | | | No  Yes |
| **6.** | **Exemption Criteria.** | | | | | Not Applicable | |
|  | A study may qualify for exemption when the only involvement of human subjects will be in one or more of the following categories (please view full exemption category / description here: https://hrpp.msu.edu/help/required/exempt-categories.html). **(*If the study does not qualify for the exemption criteria, do not complete this question and proceed to Section II.)*** | | | | | | |
| **6A.** | Exemption Categories. | | | | | | |
|  | **6A1.** | Select the category(ies) applicable to the study if the only involvement of human subjects in this study will be in one or more of the categories. Studies involving prisoners cannot be exempt UNLESS the research is aimed at involving a broader subject population that only incidentally includes prisoners *If your study is subject to U.S. Department of Justice requirements, do not complete this section; complete 6A2 below.* | | | | | |
|  |  | ***Exempt 1.*** Research conducted in established or commonly accepted educational settings, involving normal educational practices that are not likely to adversely impact students' opportunity to learn required educational content or the assessment of educators who provide instruction. ***IF YOU SELECTED THIS CATEGORY, EXPLAIN WHY THE RESEARCH WILL NOT LIKELY ADVERSELY IMPACT STUDENTS’ OPPORTUNITY TO LEARN REQUIRED EDUCATIONAL CONTENT OR THE ASSESSEMENT OF EDUCATORS WHO PROVIDE INSTRUCTION.*** | | | | | |
|  |  |  | | | | | |
|  |  | ***Exempt 2.*** Research that only includes interactions involving educational tests (cognitive, diagnostic, aptitude, achievement), survey procedures, interview procedures, or observation of public behavior. ***IF YOU SELECTED THIS CATEGORY, SELECT THE APPROPRIATE OPTION(S) BELOW.*** | | | | | |
|  |  |  | (i) Information obtained is recorded by investigator in manner that identity of subjects cannot readily be ascertained, directly or through identifiers linked to subjects | | | | |
|  |  |  | (ii) Any disclosure of subjects' responses outside research would not reasonably place subjects at risk of criminal or civil liability or be damaging to subjects' financial standing, employability, educational advancement, or reputation. | | | | |
|  |  |  | (iii) **LIMITED IRB REVIEW REQUIRED**. Information obtained is recorded by investigator in manner that identity of subjects can readily be ascertained, directly or through identifiers linked to subjects, and responses could reasonable place subjects at risk of criminal or civil liability or be damaging to the subjects' financial standing, employability, educational advancement, or reputation ***(LIMITED IRB REVIEW IS REQUIRED; YOU MUST ALSO COMPLETE QUESTION 6E TO DESCRIBE PRIVACY AND CONFIDENTIALITY SAFEGUARDS.)*** | | | | |
|  |  | ***Exempt 3.*** Research involving benign behavioral interventions in conjunction with the collection of information from an adult subject through verbal or written responses (including data entry) or audiovisual recording if the subject prospectively agrees to the intervention and information collection. ***IF YOU SELECTED THIS CATEGORY, SELECT THE APPROPRIATE OPTION(S) BELOW.*** | | | | | |
|  |  |  | (i) Information obtained is recorded by investigator in manner that identity of subjects cannot readily be ascertained, directly or through identifiers linked to subjects. | | | | |
|  |  |  | (ii) Any disclosure of subjects' responses outside research would not reasonably place subjects at risk of criminal or civil liability or be damaging to subjects' financial standing, employability, educational advancement, or reputation | | | | |
|  |  |  | (iii) **LIMITED IRB REVIEW REQUIRED**. Information obtained is recorded by investigator in manner that identity of subjects can readily be ascertained, directly or through identifiers linked to subjects, and responses could reasonable place subjects at risk of criminal or civil liability or be damaging to subjects' financial standing, employability, educational advancement, or reputation ***(LIMITED IRB REVIEW IS REQUIRED; YOU MUST ALSO COMPLETE QUESTIONS 6E TO DESCRIBE PRIVACY AND CONFIDENTIALITY SAFEGUARDS.)*** | | | | |
|  |  | ***Exempt 4.*** Secondary research uses of identifiable private information or identifiable biospecimens. ***IF YOU SELECTED THIS CATEGORY, SELECT THE APPROPRIATE OPTION(S) BELOW.*** | | | | | |
|  |  |  | Identifiable private information or identifiable biospecimens are publicly available. | | | | |
|  |  |  | Information, which may include information about biospecimens, is recorded by the investigator in such a manner that the identity of the human subjects cannot readily be ascertained directly or through identifiers linked to the subjects, the investigator does not contact the subjects, and the investigator will not re-identify subjects. ***IF YOU SELECTED THIS CATEGORY, CONFIRM THE FOLLOWING:*** | | | | |
|  |  |  |  | Investigator and research team will not contact the subjects  Investigator and research team will not re-identify the subjects | | | |
|  |  |  | The research involves only information collection and analysis involving the investigator's use of identifiable health information when that use is regulated under the Health Insurance Portability and Accountability Act (HIPAA) 45 CFR parts 160 and 164. | | | | |
|  |  |  | The research is conducted by, or on behalf of, a Federal department or agency using government-generated or government-collected information obtained for nonresearch activities, if the research generates identifiable private information that is or will be maintained on information technology that is subject to and in compliance with specific federal privacy standards. | | | | |
|  |  | ***Exempt 5.*** Federal demonstration projects. | | | | | |
|  |  | ***Exempt 6.*** Taste and food quality evaluation and consumer acceptance studies. | | | | | |
|  |  | ***Exempt 97***. ONLY applicable to research NOT FUNDED by a federal department or agency: Research involving the study of previously collected identifiable data (please view additional exclusions before selecting this category). | | | | | |
|  |  | *By checking the boxes below, you are confirming that the study will not include any of the following exclusions for the study’s duration:*  Federal funding or federal training grants  FDA regulated  Sponsor or other contractual restrictions  Clinical interventions (including clinical behavioral interventions)  Receipt of an NIH issued certificate of confidentiality to protect identifiable research data  Multi-site collaborative research study where another institution plans to rely or is relying upon MSU’s IRB review | | | | | |
|  |  | ***Exempt 98.*** ONLY applicable to research NOT FUNDED by a federal department or agency: Prospective data collection with adults through verbal or written responses involving a benign intervention (please view additional exclusions before selecting this category). | | | | | |
|  |  | *By checking the boxes below, you are confirming that the study will not include any of the following exclusions for the study’s duration:*  Federal funding or federal training grants  FDA regulated  Sponsor or other contractual restrictions  Clinical interventions (including clinical behavioral interventions)  Receipt of an NIH issued certificate of confidentiality to protect identifiable research data  Multi-site collaborative research study where another institution plans to rely or is relying upon MSU’s IRB review  Children as research subjects | | | | | |
|  | **6A2.** | **DEPARTMENT OF JUSTICE Exemption Categories*.*** Complete this section ONLY if the research is subject to Department of Justice requirements. | | | | | |
|  | **6A2i.** | Select the category(ies) applicable to the study if the only involvement of human subjects in this study will be in one or more of the categories. Studies involving prisoners cannot be exempt. | | | | | |
|  |  | ***Exempt 1.*** Research conducted in established or commonly accepted educational settings, involving normal educational practices. | | | | | |
|  |  | ***Exempt 2.*** Educational tests, survey procedures, interview procedures, observation of public behavior unless data is recorded in a manner such that subjects are identifiable and the responses could reasonably place the subjects at risk of criminal or civil liability or be damaging to the subjects’ financial standing, employability, or reputation (research cannot involve children, except for educational tests or observation of public behavior where the investigator does not interact with the child). | | | | | |
|  |  | ***Exempt 3.*** Educational tests, survey procedures, interview procedures, or observation of public behavior not otherwise exempt that involves public officials or federal statute. | | | | | |
|  |  | ***Exempt 4.*** Collection or study of existing data, documents, records, pathological specimens, or diagnostic specimens if publicly available or information is recorded by investigator in a manner that subjects cannot be identified. | | | | | |
|  |  | ***Exempt 5.*** Federal demonstration projects. | | | | | |
|  |  | ***Exempt 6.*** Taste and food quality evaluation and consumer acceptance studies. | | | | | |
|  | **6A2ii.** | Explain why the study presents minimal risk to subjects. | | | | | |
|  |  |  | | | | | |
| **6B.** | By checking the boxes below, you are confirming that the following are true and will remain true for the study’s duration: | | | | | | |
|  | Selection of subjects is equitable (considering the purposes of the research, setting in which research will be conducted, any vulnerable populations).  If there is recording of identifiable information, there are adequate provisions to maintain the confidentiality of the data.  There are adequate provisions to maintain the privacy interests of subjects.  Safeguards are or will be put in place to protect against any coercion or undue influence if you or members of your study team are or may be associated with the subjects at any point in the study (e.g. students, employees, colleagues, patients). | | | | | | |
| **6C.** | Consent | | | | | | |
| **6Ci.** | There will be a consent process for the study’s duration that will disclose information such as that the activity involves research, a description of the procedures, that participation is voluntary and withdrawal is without penalty, and the name and contact information for the researcher (select appropriate option below): | | | | | | |
|  | For All Subjects  For Some Subjects  For None of the Subjects (consent will not be obtained)  *CLICK IRB: Upload the consent document to the Consent Forms and Recruitment Materials SmartForm page.* | | | | | | |
| **6Cii.** | Please explain your selection. | | | | | | |
|  | Consent will be obtained for all participants. | | | | | | |
| **6D.** | Please acknowledge that you may not begin the research at non-MSU institutions (regardless of engagement), until you receive the appropriate approvals/permissions from the sites (e.g. IRB review/exempt determination from non-MSU sites, data use or research agreements, other regulatory approvals). An MSU exempt determination does not provide approval/permission for a non-MSU site, including sites with reliance agreements with MSU. Please note that non-MSU sites may have requirements that differ from MSU for exempt research. Note that this also applies to sites added after the MSU exempt determination. | | | | | | |
|  | Acknowledged | | | | | | |
| **6E.** | **LIMITED IRB REVIEW.** If the exemption(s) require limited IRB review (if you selected Exemption 2(iii) or 3(i)(C) in Question 6A), complete questions 1 and 2 to describe privacy and confidentiality. | | | | | | |
| **6E1.** | **Privacy of Subjects.** | | | | | | |
|  | How will subjects’ privacy be protected? Consider the number of individuals interacting with the subject or subject’s records, location of consent process and study, presence of individuals not associated with the study, sensitivity of the research. | | | | | | |
|  | The information we will collect is only for the study and will NOT be shared with the YWCA, OR Hurley Medical Center. We are committed to protecting your privacy. | | | | | | |
| **6E2.** | **Confidentiality of Data.** | | | | | | |
| **6E2i.** | Select the appropriate option: | | | | | | |
|  | Identifying or coded information will not be stored with the information and/or biospecimen(s)  Identifying or coded information will be stored with the information and/or biospecimen(s) | | | | | | |
| **6E2ii.** | Please explain your selection. If you are storing identifying or coded information with the information and/or biospecimen(s), explain why identifiable or coded information and/or biospecimen(s) needs to be maintained and how long it will be necessary to maintain it. | | | | | | |
|  | N/A-- identifiable information will not be stored with the data. | | | | | | |
| **6E2iii.** | Describe the procedures and safeguards you will use to secure the information and/or biospecimen(s), including during transport of information and/or biospecimen(s). | | | | | | |
|  | We will minimize potential risks due to loss of confidentiality of research data by having all  information collected and handled by research staff, including study mentor mothers, trained to deal appropriately with sensitive clinical issues. All participants will be informed about the limits of confidentiality concerning suicidal intent, homicidal intent, suspected child abuse, and suspected elder abuse. All information will be treated as confidential material and will be available only to research staff. All information will be kept in locked file cabinets. Computer data files will be available only to authorized personnel and no names or obvious identifying information will be stored in data files. No participant will be identified in any report of the  project. Participants will update their contact information and contact person at each assessment point to ensure that this information remains accurate. No information about participants will be released without their permission or where required by law. Participants will update their contact information and contact person for collateral interviews at each follow-up interview to ensure that this information remains appropriate. Audio recording of assessments and mentor mother conversations is necessary to ensure fidelity to established protocols. This is accomplished through the use of encrypted credit-card size digital audio  recorders. These digital recordings will be transferred to Michigan State University’s secure computer server (designed to hold and protect digital audio recordings for clinical trials) by mentor mothers and research assistants via secure server access. Participants will be asked to give informed written consent to audio recording at the time of intervention study entry. Per the consent form, participants understand that most mentor mother sessions are audio  recorded, but mentor mothers and research assistants always say “are you comfortable with recording today?” and if the participant says, “no,” the session will not be recorded. We will modify this process based on the results from the open trial. We may switch to checklists if its use in open trial doesn’t support feasibility. To assure the confidentiality and protection of participants with respect to audio taping, the following steps will be taken. a) each recording will be labeled with the participant’s study identification number, the intervention provider’s name, and the session date; b) all recordings will be stored on a secured computer server designed to hold and protect research data; c) access to the audio recordings will be limited to specially trained research assistant. The research assistant will rate the recordings for adherence and competence. Study PI and Co-I’s will provide ongoing guidance regarding the conduct and design of the intervention; and d) we will use encrypted audio recorders.  We will also minimize the risk of breach of confidentiality by the mentor mothers. All mentor mothers will receive MSU’s human subjects training. As is standard practice in paraprofessional delivered interventions, we will emphasize the importance of protecting confidentiality at the beginning and throughout the intervention process. Mentor mothers will be informed at the beginning of the intervention that any substantiated complaint of breach of confidentiality will result in possible termination of their contract. | | | | | | |
|  |  | | | | | | |

| **Other Click IRB Documents to Upload As Appropriate**  **(Applicable to All Studies)** |
| --- |
| - *Upload this completed protocol to the Basic Information SmartForm page, Question 10.* - *Upload any funding materials not accessible in Kuali Coeus in the Supporting Documents SmartForm page.* - *Upload the HRP-537 - Template - Use of Protected Health Information Application to the MSU Additional Study Information SmartForm page.* - *Upload the HRP-538 - Template - MSU Authorization to Use or Disclose Health Information for Researchers to the MSU Additional Study Information SmartForm page.* |
|  |
| **IF THE STUDY MAY QUALIFY FOR AN EXEMPTION**  **(INCLUDING THOSE THAT MAY REQUIRE LIMITED IRB REVIEW),**  **STOP HERE AND DO NOT COMPLETE SECTION II.**  **CONTINUE ONLY IF THE STUDY**  **DOES NOT QUALIFY FOR AN EXEMPTION.**  **COMPLETE QUESTIONS 7-23 FOR**  **AN EXPEDITED OR FULL BOARD STUDY.** |

| **Section II. Additional Questions for an Expedited or Full Board Study**  Not all questions or sections are applicable to every study. If the question or section is not applicable, check the “Not Applicable” box. All other questions are required. | | | | | | | | |
| --- | --- | --- | --- | --- | --- | --- | --- | --- |
| **7.** | **Expedited Categories.** | | | | | | | |
| **7A.** | Please select the Expedited category(ies) and sub-categories as applicable to the study if the only involvement of human subjects in this study will be in one or more of the categories. If the study involves more than minimal risk or none apply, select “The study involves more than minimal risk OR none of the expedited category(ies) apply.” | | | | | | | |
|  |  | ***The study involves more than minimal risk OR none of the expedited categories apply. IF THIS OPTION IS SELECTED, DO NOT SELECT ANY OF THE EXPEDITED CATEGORY(IES).*** | | | | | | |
|  |  | ***Expedited 1*.** Clinical studies of drugs and medical devices only when condition (a) or (b) is met. ***IF YOU SELECTED THIS CATEGORY, SELECT THE APPROPRIATE OPTION(S) BELOW.*** | | | | | | |
|  |  |  | (a) Research on drugs for which an investigational new drug application (21 CFR Part 312) is not required. (Note: Research on marketed drugs that significantly increases the risks or decreases the acceptability of the risks associated with the use of the product is not eligible for expedited review.) | | | | | |
|  |  |  | (b) Research on medical devices for which (i) an investigational device exemption application (21 CFR Part 812) is not required; or (ii) the medical device is cleared/approved for marketing and the medical device is being used in accordance with its cleared/approved labeling. | | | | | |
|  |  | ***Expedited 2*.** Collection of blood samples by finger stick, heel stick, ear stick, or venipuncture. ***IF YOU SELECTED THIS CATEGORY, SELECT THE APPROPRIATE OPTION(S) BELOW.*** | | | | | | |
|  |  |  | (a) from healthy, nonpregnant adults who weigh at least 110 pounds. For these subjects, the amounts drawn may not exceed 550 ml in an 8 week period and collection may not occur more frequently than 2 times per week; or | | | | | |
|  |  |  | (b) from other adults and children [2], considering the age, weight, and health of the subjects, the collection procedure, the amount of blood to be collected, and the frequency with which it will be collected. For these subjects, the amount drawn may not exceed the lesser of 50 ml or 3 ml per kg in an 8 week period and collection may not occur more frequently than 2 times per week | | | | | |
|  |  | ***Expedited 3***. Prospective collection of biological specimens for research purposes by noninvasive means. | | | | | | |
|  |  | ***Expedited 4***. Collection of data through noninvasive procedures (not involving general anesthesia or sedation) routinely employed in clinical practice, excluding procedures involving x-rays or microwaves. Where medical devices are employed, they must be cleared/approved for marketing. (Studies intended to evaluate the safety and effectiveness of the medical device are not generally eligible for expedited review, including studies of cleared medical devices for new indications.) | | | | | | |
|  |  | ***Expedited 5***. Research involving materials (data, documents, records, or specimens) that have been collected, or will be collected solely for nonresearch purposes (such as medical treatment or diagnosis). | | | | | | |
|  |  | ***Expedited 6***. Collection of data from voice, video, digital, or image recordings made for research purposes. | | | | | | |
|  |  | ***Expedited 7***. Research on individual or group characteristics or behavior (including, but not limited to, research on perception, cognition, motivation, identity, language, communication, cultural beliefs or practices, and social behavior) or research employing survey, interview, oral history, focus group, program evaluation, human factors evaluation, or quality assurance methodologies. | | | | | | |
| **7B.** | **For Studies Regulated by the U.S. Food and Drug Administration or the U.S. Department of Justice**. If you selected an expedited category, explain why the study presents minimal risk to subjects. | | | | | | | |
|  |  | | | | | | | |
| **8.** | **More than Minimal Risk Research.** *Complete the following question if you selected “The study involves more than minimal risk OR none of the expedited categories apply” in Question 7A (Expedited Categories).* | | | | | | | |
| **8A.** | Describe the relevant prior experience and gaps in current knowledge, relevant preliminary data, if any, and the scholarly background for, and significance of, the research based on existing literature and how it will add to existing knowledge. | | | | | | | |
|  |  | | | | | | | |
| **8B.** | Sample Size. | | | | | | | |
| **8Bi.** | Total number of subjects who will be approached (including screen failures, controls and subject withdrawals) to reach enrollment numbers for the lifetime of the study at this investigator’s sites. | | | | | | | |
|  | 40 | | | | | | | |
| **8Bii.** | Total number of subjects who will be enrolled in the study at this investigator’s site. | | | | | | | |
|  | 40 | | | | | | | |
| **8Biii.** | Describe the statistical justification or rationale for the proposed sample size. Considerations for sample size may include the acceptable level of significance, power of the study, expected effect size, underlying event rate in the population, standard deviation in the population, saturation of themes, and/or have a theoretical basis. | | | | | | | |
|  | The purpose of the R34 Exploratory Research Award is to collect preliminary data to assess  feasibility and acceptability of an intervention and to inform a larger RCT. With data from 40  participants in intent to treat analyses (about 20 per condition), we would only have statistical  power adequate (.80) to detect large effects (d = .91) with alpha of .05. Our primary  emphasis will be on examining the direction of effects and the range of effect sizes for  differences between conditions. All estimates of effect size resulting from this study will  include 95% confidence intervals. With 10% study attrition for this trial, the study will have  complete data on 36 individuals. Power for a 2-tailed t-test at alpha = .05 for 18 individuals in  each condition is 30% for an effect size of d = .5. If study attrition is higher than expected  (i.e., 18%), the study has similar power (28% for d = .5). Although we will be examining 95%  confidence intervals around effects, per NIMH and Helena Kraemer1, the primary purpose of  R34 pilot studies is to develop interventions and test feasibility/acceptability of interventions  and research procedures. | | | | | | | |
| **9.** | **Minimal Risk Research.** *Complete the following question if you selected an expedited category in Question 7A.* | | | | | | | |
| **9A.** | Briefly describe the background for conducting the research. (1-2 sentences) | | | | | | | |
|  | The study will provide mentoring intervention for pregnant women and mothers with children under 5 who report experience of IPV and elevated levels of PTSD and depressive symptoms. | | | | | | | |
| **9B.** | Sample Size. | | | | | | | |
| **9Bi.** | Provide an estimated sample size for the lifetime of the study at this investigator’s sites. | | | | | | | |
|  | 40 | | | | | | | |
| **9Bii.** | Describe the basis for that estimate. | | | | | | | |
|  | For this R34 services development grant, assessment of feasibility and acceptability of the  intervention and research procedures is the primary goal. In terms of other outcomes, we are  well aware that effect size estimates with small samples have large standard errors and therefore wide confidence intervals2 (NIMH PAR-09-173) and believe that the most important factor in determining sample size for a Stage II clinical trial is for that trial to have adequate power to detect a clinically relevant difference between conditions if one truly exists. Nonetheless, pilot data can be used to demonstrate whether the effects of treatment look promising across a set of outcome variables, to begin to examine distribution of outcome variables to inform future analytic strategies, and to suggest, in concert with results from larger scale clinical trials in related fields, the range of effect sizes that would be reasonable to expect in a future trial. As a result, we will obtain the between treatment condition effect size estimates (with 95% confidence intervals) at each assessment (e.g., Cohen’s d or h) as well as the correlation between the same dependent variable at adjacent assessments. These parameters will provide one source of information that will be used to help determine necessary sample size for a future clinical trial. We will have complete data on approximately 40 participants (the minimum expected after participant loss due to attrition). Sample size guidelines for treatment development from Rounsaville et al.3 recommend 15 to 30 participants per cell. Given that group means begin to stabilize by around 15, we believe a sample of 20 in each condition should provide some information relevant to demonstrating potential promise for the MOSAIC Plus intervention.  Primary analyses will be intent-to-treat (using data from all treatment enrollees). We will also conduct secondary dose-response analyses. Analysis strategies used (hierarchical linear modelling [HLM]) can accommodate missing data, and can be used in a sample of 40 with a highly constrained covariance structure.  Data Checking: Standard data checking procedures will include checking forms for missing data, double entry with discrepancy resolution, daily back-up copies of computer files, and examination of key variables for skewness, variability, missing data, and outliers. Data will be transformed to achieve normality if needed. If any outcome variable is too zero inflated to be normalized through transformation, it will be analyzed with logistic regression or generalized estimating equation techniques in this small pilot study, and with zero-inflated Poisson or negative binomial regression techniques in the subsequent fully-powered study. Per clinical trials guidelines4, analyses will adjust for baseline levels of dependent variables but will not test for or adjust for any other baseline differences between conditions that result  from randomization.  Study Feasibility and Treatment Feasibility/Acceptability. One of the primary goals of a treatment development study is to demonstrate the feasibility of the proposed treatment and of the study and recruitment methods2. As a result, we will assess the feasibility of the research procedures by examining study recruitment and refusal rates, participants’ willingness to be randomized, follow-up rates, reliability and range of responses to study questionnaires, and success of the MOSAIC Plus mentor mothers training program. We will assess the feasibility and acceptability of the MOSAIC Plus intervention by examining rates of treatment attendance, rates of treatment completion (based on the jointly established treatment plan) and drop-out, and scores on the End of Treatment Questionnaire. We will also examine reasons for termination for consistent patterns. We will examine the acceptability of the MOSAIC Plus mentoring intervention by using data from CTQ-8 treatment satisfaction questionnaire and detailed exit interviews. Perinatal women’s experiences with the MOSAIC Plus mentor mothers, the quality of their working relationship and their level of satisfaction with the service will evaluated using the Working Alliance  Inventory-Short Revised (WAI-SR)5. | | | | | | | |
| **10.** | **Benefits.** | | | | | | | |
|  | Describe any potential direct benefit(s) to subjects in this study, if any and the importance of the knowledge that may reasonably be expected to result. Within the description, do not include payment to subjects as a benefit. | | | | | | | |
|  | If a participant is matched to the MOSAIC Plus group, the support they will receive from the mentor mother may help them build and strengthen your social support networks, identify and access community mental health, medical care and other services they may need. If participants are assigned to the MOSAIC group, they will receive MOSAIC mentoring support from a study mentor mother. There are also other services that have nothing to do with this study. We will give participants a list of treatment resources in your community. During 3 and 6 months assessments, participants will receive $50 for their time compensation. | | | | | | | |
| **11.** | **Screening, Recruitment, and Determining Eligibility.** | | | | | | | |
| **11A.** | Describe how subjects will be identified and recruited, including who will perform the recruitment. | | | | | | | |
|  | Recruitment will take place at the Obstetrics and Gynecological Unit of Hurley Medical Center in Flint, Michigan and at the YWCA Flint. Hurley Medical Center serves approximately 3,000 births each year, ranging from textbook pregnancies to high-risk deliveries. The average monthly OB intake is approximately around 300-350 new pregnancies. The YWCA Flint serves ~270 women per month as part of their sexual abuse and domestic violence program. Majority of these women are pregnant and/or mothers with young children. Ratios in the expected inclusion enrollment report reflect the Genesee County population; (72.3% white, 19.6% African-American, 3.36% Hispanic and others 4%). We will enroll on the average 4 individuals (who meet the study criteria and consent to participate) per month for the open trial (with a sample of 15) and the randomized trial (a sample of 40) starting from months 9 and 19 respectively. Recruitment of participants for the open trial and randomized trial will take 3 and 10 months respectively. The study research assistant will consent study participants, do assessments and introduce them to the study mentor mothers. | | | | | | | |
|  | *CLICK IRB: Upload the recruitment materials to the Consent Forms and Recruitment Materials SmartForm page, Question 2.* | | | | | | | |
| **11B.** | The study team will obtain for the purpose of screening, recruiting, or determining the eligibility of prospective subjects (please select the appropriate option(s)): | | | | Not Applicable | | | |
|  | Information through oral or written communication with the prospective subject or legally authorized representative. Before the information is obtained for the purpose of screening, recruiting, or determining eligibility, consent:  will be obtained.  will not be obtained. *Please describe screening consent procedures in Question 12.* | | | | | | | |
|  | Identifiable private information or identifiable biospecimens by accessing records or stored identifiable biospecimens. Before the information is obtained for the purpose of screening, recruiting, or determining eligibility, consent:  will be obtained.  will not be obtained. *Please describe screening consent procedures in Question 12.*  *Note: The revised Common Rule permits an exception from informed consent for screening, recruiting, or determining eligibility when certain criteria are met; this exception does not apply to studies subject to the Pre-2018 Common Rule Requirements and/or studies regulated by the U.S. Food and Drug Administration (FDA).* | | | | | | | |
| **11B1.** | Please explain your selection(s). | | | | | | | |
|  | The study research assistant will screen participants by interviewingwomen at the YWCA-Flint Shelter or at Hurley Medical Center at the OBGYN clinic. | | | | | | | |
| **12.** | **Consent Process.** | | | | | | | |
| **12A.** | If the study involves adults, consent will be obtained from (select appropriate option(s)): | | | | | Not Applicable | | |
|  | All subjects  Some subjects  No subjects (consent will not be obtained) | | | | | | | |
|  | *CLICK IRB: Upload the consent document, script, etc. (including translations) to the Consent Forms and Recruitment Materials SmartForm page, Question 1.* | | | | | | | |
| **12B.** | If the study involves children, parental permission will be obtained from (select appropriate option(s)): | | | | | Not Applicable | | |
|  | Both parents or guardians (unless one parent is deceased, unknown, incompetent, or not reasonably available, or when only one parent has legal responsibility for the care and custody of the child)  One parent or guardian  Will not be obtained | | | | | | | |
|  | *CLICK IRB: Upload the parental permission forms to the Consent Forms and Recruitment Materials SmartForm page, Question 1.* | | | | | | | |
| **12C.** | If the study involves children, child assent will be obtained from (select appropriate option): | | | | | Not Applicable | | |
|  | All children  Some children  Will not be obtained | | | | | | | |
|  | *CLICK IRB: Upload the child assent form to the Consent Forms and Recruitment Materials SmartForm page, Question 1.* | | | | | | | |
| **12D.** | Describe the consent process, including an explanation of your selection(s) above. If the study involves screening activities, please describe whether consent will be obtained and if consent will not be obtained, explain how the screening data will be used. If only some subjects will provide consent, explain who will or will not provide consent. If only some children will provide assent, explain which children will and will not provide assent. | | | | | | | |
|  |  | | | | | | | |
| **12E.** | If consent will not be obtained, explain why. Describe why the research could not be practicably carried out if consent was required. If the research involves identifiable private information or identifiable biospecimens, describe why the research could not practicably be carried out without using such information or biospecimens in an identifiable format. | | | | | | | Not Applicable |
|  |  | | | | | | | |
| **12F.** | If your study involves use of a consent form, complete i, ii, and iii. | | | | | | | Not Applicable |
| **12Fi.** | Select the appropriate option(s) below for the documentation of consent.  Will use a written consent document signed by subjects  Will use a short form written consent document signed by subjects  Will not obtain a signed consent document for some subjects  Will not obtain a signed consent document for all subjects | | | | | | | |
| **12Fii.** | Describe when and how the subject will receive a copy of the consent form. | | | | | | | |
|  | The study research assistant will provide a copy of the consent form for the participant prior to screening and assessments. | | | | | | | |
| **12Fiii.** | If subjects will not be signing the consent document, please explain why. If some subjects will not sign the consent document, explain who will and will not sign the consent. | | | | | | | Not Applicable |
|  |  | | | | | | | |
| **12G.** | If the study involves cognitively impaired adults, explain the process to determine whether a subject is capable of consent, use of any legally authorized representative(s), and any assent process. | | | | | | | Not Applicable |
|  |  | | | | | | | |
|  | *CLICK IRB: Upload any assessment tools to the Supporting Documents SmartForm page.* | | | | | | | |
| **13.** | **Coercion or Undue Influence.** | | | | | | | |
| **13A.** | If some or all of the subjects are likely to be vulnerable to coercion or undue influence, such as children, prisoners, pregnant women, mentally disabled persons, individuals with impaired decision-making capacity, or economically or educationally disadvantaged persons, describe additional safeguards that have been included in the study. | | | | | | | Not Applicable |
|  | We will minimize the risk of potential coercion by following standard procedures for obtaining informed consent. We will begin this process during the intake, where we will clarify the nature of the study and possible alternatives from the outset. Prior to enrolling women in the research, we will fully explain the study procedures, risks, benefits, and alternatives to women, emphasizing that the woman’s participation has no impact on the other services she receives at the community or at the health facility. Also, women who do not consent or who withdraw at any point during the study will receive appropriate referrals (e.g., mental health, perinatal care and/or substance use treatment), if needed. All women will be reminded that there is no penalty for women who choose to not participate or to withdraw from the study and that their decision to participate does not impact the standard services they receive through the OB unit of Hurley Medical Center, YWCA or at the community. All reimbursements for participating will be commensurate with participants’ time required for participating in the research. | | | | | | | |
| **13B.** | If you or your study team are associated with the subjects (e.g. your students, employees, colleagues, patients), explain the nature of any association and measures taken to protect subjects’ rights, including safeguards against any coercion or undue influence (e.g. pressure a subject might feel to participate based on the association). | | | | | | | Not Applicable |
|  |  | | | | | | | |
| **14.** | **Privacy.** | | | | | | |  |
|  | How will subjects’ privacy be protected? Consider the number of individuals interacting with the subject or subject’s records, location of consent process and study, presence of individuals not associated with the study, sensitivity of the research. | | | | | | | |
|  | Only trained study staff and mentor mothers will interact with the study participants. Both Hurley Medical Center and the YWCA-Flint will provide a private room to conduct study screening and assessments. No other individual would be present during study assessments. | | | | | | | |
| **15.** | **Withdrawal of Subjects.** | | | | | | | Not Applicable |
|  | If there are any anticipated circumstances where the researcher will withdraw subjects from the study regardless of the subject’s wishes, describe the circumstances and the procedures when subjects are withdrawn from the study. | | | | | | |  |
|  |  | | | | | | | |
| **16.** | **Monitoring Plan to Assess Data to Ensure Safety of Subjects.** | | | | | | |  |
| **16A.** | Is there a monitoring plan to periodically assess the data to ensure the safety of subjects or to ensure negative outcomes do not occur? | | | | | | | No  Yes |
|  | Explain your answer. If you answered Yes, describe the monitoring plan. | | | | | | |  |
|  | Data Monitoring Plan  The main interest of this study is to test the feasibility and acceptability of the intervention methods, training procedures, approaches for recruitment and retention of participants and dropouts and rates of completion. Other outcomes of interest of this study comparing MOSAIC Plus to ETAU include reduction in depressive and PTSD symptoms, decreased subsequent IPV experience over 9 months, improvement in functioning self-care and general health and wellbeing. As mechanisms, we will evaluate social support provided by mentor mothers will increase their access to social support networks and enhance their effectiveness in obtaining resources.  An experienced team will conduct data management and data entry. The team will establish a participant tracking and data monitoring system. The research assistant will be trained in interviewer-administered instruments (ETQ, MOS-SS, CSQ, PHQ-9, DTS, CTS- 2, WHODAS-II, SF-36, etc.) to complete follow-up assessments; fidelity to assessments will be monitored regularly. Baseline data will be collected using standardized paper forms and will only be identified with the study’s ID of the participant. The codes that link the name of the participant and the study ID will be kept confidential in secured cabinets at MSU. Collected forms will be transported to the PI’s data entry center at MSU. Surveys are administered using a password-protected web server administration web page. The assessment survey software and identification databases are linked using a subject ID number that is assigned upon enrollment into the project. All data collected from participants is coded using that number.  Data stored on the server are password-protected, and are downloaded to desktop computers belonging only to authorized study personnel. Neither data nor reports contain any identifying information. All mentoring sessions will be recorded using encrypted, password-enabled credit-card sized digital audio recorders that are effective for quality recording and portable. Mentor mothers then will upload the recordings to our secure research audio server from their (remote) computers by using WinSCP. Data quality will be monitored by random inspection of completed forms and databases by Dr.  Hailemariam and any problems detected will be discussed with the Co-Is. Standard data checking procedures will include checking forms for missing data, double entry with discrepancy resolution, daily back-up copies of computer files, and examination of key variables for skewness, variability, missing data, and outliers. The computer systems used for data entry and analysis are protected by passwords and secure login and data communications procedures to minimize the potential for disclosure of research information either inadvertently or as a result of external attack. Within each computer system, only those users authorized to access the data for a given study are able to do so. Research records are stored in areas that are locked when staff is not present.  Safety monitoring plan  All unanticipated problems related to participation in the study will be reported to the PI or delegated research staff. The PI will be the key responsible person in identifying potential unanticipated problems, adverse events (AEs) and Serious Adverse Events (SAEs) encountered by the study participants. She will make modifications/adaptations to the intervention to address the issue and will oversee reporting. Reports of unanticipated problems will be submitted to the respective IRBs and NIH within the required timeframe. The NIH definitions of AEs and Serious Adverse Events SAEs for clinical research will be used. When a member of the research staff is notified an AE or SAE, she or he will immediately document the date and details of the event in the Adverse Event Tracking Log. All reporting procedures described below will also be implemented as required. Specific categories of problems that require additional action are detailed below.  The PI, Dr. Hailemariam, will be responsible for overseeing the safety of all participants.  Participant safety will be monitored in two ways: (a) during the intake or ongoing assessments by the research staff, or (b) during intervention sessions. Research assessments will be conducted at baseline, 3, 6 and 9 month follow-up. During these assessments, we will monitor all participants for significant suicidal ideation (SI), clinical deterioration, and study related partner violence. We also describe procedures for reporting child abuse, addressing homicide ideation, and monitoring the safety of mentor mothers. | | | | | | | |
|  | *CLICK IRB: Upload any data safety monitoring plans to the Supporting Documents SmartForm pages.* | | | | | | | |
| **16B.** | If there is a data safety monitoring committee or board, describe the composition and frequency of meetings. | | | | | | | Not Applicable |
|  | An external local Data and Safety Monitoring Board (DSMB) will be assembled to evaluate the data and safety to participants enrolled in the study. We will recruit 3 non-MSU-affiliated board members who have experience in clinical trials and/or research involving women or other high-risk populations as well as the ethical issues involved with a randomized control study. Recruiting these researchers wouldn’t be difficult because our faculty is located strategically where many other universities including University of Michigan, and numerous other universities and colleges in Michigan are operating.  The external DSMB will convene twice in Year 1, and then at the end of each year in Years 2-3 for a meeting. Initially, the Board will convene with the PI to review the study protocol and review the guidelines for data and safety monitoring. This will include establishing standard procedures for daily (whenever there has been contact with a subject) and weekly monitoring by the local internal reviewers (PI and study personnel). At this meeting and at each subsequent meeting, the DSMB will evaluate recruitment, the progress of the trial, subject retention, data quality and confidentiality. In addition, they will review subjects' clinical status, rates of adverse events and whether or not there have been any changes in risk to participating subjects. This review will ensure that subject risk does not outweigh study benefits. In the DSMB’s review of adverse events, if non-serious adverse events are occurring at a significantly higher rate in one condition than the other, then the DSMB will make appropriate recommendations for changes in the protocol, if needed. If Serious Adverse Events (SAEs) occur at a significantly higher rate in one condition than the other, then the DSMB might consider terminating the trial, if changes to the protocol are unlikely to address the high occurrence of the SAEs. We do not anticipate that this will occur, because we have taken several steps to avoid or protect against the occurrence of SAEs as outlined in the section on safety monitoring plan. A report generated from each of these meetings will be retained at the study site and will be forwarded to the university IRB and to NIMH.  The DSMB will be available to convene outside of the appointed meeting schedule, if necessary, due to concerns regarding a particular subject, or due to any troublesome developments in subjects' experiences during the study. The DSMB will make appropriate recommendations for changes in the study protocol, if needed.  The safety of participants will be monitored during each contact with the research team. Both  anticipated and unanticipated adverse events and problems will be formally monitored and recorded. Unanticipated serious adverse events or problems will be reported to the university IRB and to NIMH within required reporting timeframes. Anticipated and less serious adverse events will be submitted annually in reports to both the IRBs and NIMH.  The study PI will be responsible for monitoring the safety and efficacy of this trial, executing the Data and Safety Monitoring Plan (DSMP) and complying with the reporting requirements. The PI will provide a summary of the DSMB’s report to NIMH on an annual basis as part of the progress report. The DSMB report will include the participants’ sociodemographic characteristics, treatment retention rates, any quality assurance or regulatory issues that occurred during the past year, summary of adverse events and serious adverse events, and any actions or changes with respect to the protocol. The DSMB report to NIMH will also include, when available, the results of any feasibility, acceptability, or other data analysis conducted. | | | | | | | |
| **17.** | **Results and Data Sharing.** | | | | | | |  |
| **17A.** | Could this research generate any results that could be clinically relevant, including individual research results, or general, or aggregate research findings? | | | | | | | |
|  | No  Yes, clinically relevant individual research results  Yes, clinically relevant general or aggregate research findings | | | | | | | |
| **17A1.** | If yes, explain what clinically relevant research results will be generated, whether they will be disclosed to subjects or others (e.g. subject’s primary care physician), and if so, under what conditions. Address individual research results and/or general or aggregate research findings, as appropriate. *This also needs to be explained in the consent document.* | | | | | | | |
|  |  | | | | | | | |
| **17B.** | For other research results, select all that apply: | | | Not Applicable | | | | |
|  | Overall study results will be shared directly with subjects  Individual results or incidental findings of individual subjects will be shared with subjects or others  Data will be submitted to a repository or database as part of data sharing agreement (e.g. genomic data sharing) | | | | | | | |
| **17B1.** | Explain your selection(s), including how the data or results will be shared and with who (e.g. subject’s primary care physician, data repository). | | | | | | | |
|  | The primary goal of this study is feasiblity and acceptability of the MOSAIC Plus intervention which may not be relevant for the subject's primary care physician. | | | | | | | |
| **18.** | **Local Context and Multi-Site Study.** | | | | | | | |
| **18A.** | Describe the locations of where the study team will obtain information or biospecimens through intervention or interaction with the subject or obtain the subjects’ private identifiable information. | | | | | | | |
|  | Participants will be recruited from Hurley Medical Center, OBGYN clinic and from YWCA Flint. | | | | | | | |
| **18B.** | If the study will engage employees or agents of non-MSU organizations (e.g. performance sites), explain how the employees or agents will be engaged (e.g. will they perform research procedures, will they obtain informed consent from subjects). | | | | | | | Not Applicable |
|  | No other employees or agentsfrom non-MSU organizations will be involved. | | | | | | | |
| **18C.** | If the study involves multiple performance sites, describe the methods for communicating with engaged sites related to the protection of human subjects (e.g. any potential unanticipated problems that may involve risks to subjects others). | | | | | | | Not Applicable |
|  | Noth sites are located in Flint and one research assistant will be in charge of both. | | | | | | | |
| **18D.** | If there are any cultural or local contexts or requirements that may impact the protection of human subjects or present additional risks to subjects that have not otherwise been described, please describe. If research is conducted outside the state of Michigan, this could include additional state or international requirements or laws. | | | | | | | Not Applicable |
|  |  | | | | | | | |
| **18E.** | If translations to a language other than English will be provided to subjects, describe the translation process. | | | | | | | Not Applicable |
|  |  | | | | | | | |
|  | *CLICK IRB: Upload translated documents to the appropriate SmartForm page(s).* | | | | | | | |
| **19.** | **Resources and Financial Compensation and Costs.** | | | | | | | |
| **19A.** | If someone will receive a payment for recruiting the subjects, explain the amount of payment, who pays it, who receives it, and why they are being paid. | | | | | | | Not Applicable |
|  |  | | | | | | | |
| **19B.** | If subjects will incur additional financial costs as a result of their participation in this study, explain the additional costs. | | | | | | | Not Applicable |
|  |  | | | | | | | |
| **19C.** | Describe any resources not otherwise described elsewhere in the submission (e.g. internal funding) for the protection of human subjects. | | | | | | | Not Applicable |
|  |  | | | | | | | |
|  | *CLICK IRB: Upload any funding materials not accessible in Kuali Coeus in the Supporting Documents SmartForm page*. | | | | | | | |
| **19D.** | If subject’s biospecimens (even if identifiers are removed) may be used for commercial profit, describe whether the subject will or will not share in the commercial profit. *This also needs to be explained in the consent document.* | | | | | | Not Applicable | |
|  |  | | | | | | | |
| **20.** | **Information and/or Biospecimen(s) Management and Confidentiality.** | | | | | | | |
| **20A.** | Select the appropriate option: | | | | | | | |
|  | Identifying or coded information will not be stored with the information and/or biospecimen(s)  Identifying or coded information will be stored with the information and/or biospecimen(s) | | | | | | | |
| **20B.** | Please explain your selection. If you are storing identifying or coded information with the information and/or biospecimen(s), explain why identifiable or coded information and/or biospecimen(s) needs to be maintained and how long it will be necessary to maintain it. | | | | | | | |
|  |  | | | | | | | |
| **20C.** | Describe the procedures and safeguards you will use to secure the information and/or biospecimen(s), including during transport of information and/or biospecimen(s). | | | | | | | |
|  | Participants will be assigned study ID numbers, and all identifiable information will be de-identified or removed. Data will be stored in a separate password protected computer. | | | | | | | |
| **21.** | **Drug and/or Device Storage, Handling, and Administration.** | | | | | | | Not Applicable |
|  | Describe the procedure and plan for storage, handling, and administration of the drug and/or device so that they will be used only on enrolled subjects and be used only by authorized study personnel. | | | | | | | |
|  |  | | | | | | | |
| **22.** | **Future Research.** | | | | | | |  |
|  | If the research involves the collection of identifiable private information or identifiable biospecimens, select the appropriate option: | | | | | | | Not Applicable |
|  | The subject’s information or biospecimens, even if identifiers are removed, could be used for future research studies or distributed to another investigator for future research studies  The subject’s information or biospecimens, even if identifiers are removed, will NOT be used or distributed for future research studies | | | | | | | |
|  | *Please be sure to carefully consider the appropriate option, as this needs to be explained in the informed consent and can limit what is done or used for future research.* | | | | | | | |
| **23.** | **MSU Additional Information.** | | | | | | | Not Applicable |
|  | Identify if your study involves any of the following: (check all that apply) | | | | | | | |
|  | Use of human stem cells  Research with biospecimens will (if known) or might include whole genome sequencing (i.e., sequencing of a human germline or somatic specimen with the intent to generate the genome or exome sequence of that specimen). *If so, this needs to be explained in the consent document.* | | | | | | | |
|  |  | | | | | | | |
|  | **Other Click IRB Document Uploads As Appropriate**  **(Applicable to Expedited or Full Board Studies)** | | | | | | | |
|  | - *Upload list of external study team members (non-MSU individuals) to the Study Team Members SmartForm page, Question 2.* - *Upload other institution(s) approval letter(s), if submitted to other IRB(s) or ethics committees, to the Supporting Documents SmartForm page.* - *Upload FDA communications, package inserts, FDA form 1572, or other information related to drugs or devices to the appropriate Drug or Device SmartForm pages.* - *Upload the HRP-540 - Template - ICH-GCP - For Investigator to the MSU Additional Study Information SmartForm page.* - *Upload HRP-541 - Template - Involvement of Prisoners in a Research Project to the MSU Additional Study Information SmartForm page.* - *Upload the investigator brochure to the Supporting Documents SmartForm page* - *Upload the MRI Screening Form – Women to the Supporting Documents SmartForm page.* - *Upload the translation of instrument(s) provided to non-English speaking subjects to the Supporting Documents SmartForm page.* - *Upload the curriculum vitae(s) when research is more than minimum risk to the Supporting Documents SmartForm page.* - *Upload case report forms to the Supporting Documents SmartForm page.* - *Upload the Non-MSU Employee Conflict of Interest Disclosure Form to the Supporting Documents SmartForm page.* - *Upload any other pertinent documents related to the proposed research study to the Supporting Documents SmartForm page* | | | | | | | |
